# Antimicrobial, Antivirulence, and Antiparasitic Potential of *Capsicum chinense* Jacq. Extracts and Their Isolated Compound Capsaicin

**DOI:** 10.3390/antibiotics11091154

**Published:** 2022-08-26

**Authors:** Ralciane de Paula Menezes, Meliza Arantes de Souza Bessa, Camila de Paula Siqueira, Samuel Cota Teixeira, Eloisa Amália Vieira Ferro, Mário Machado Martins, Luis Carlos Scalon Cunha, Carlos Henrique Gomes Martins

**Affiliations:** 1 Technical School of Health, Federal University of Uberlândia, Uberlândia 38400-732, MG, Brazil; 2 Laboratory of Antimicrobial Testing, Federal University of Uberlândia, Uberlândia 38400-732, MG, Brazil; 3 Laboratory of Immunophysiology of Reproduction, Institute of Biomedical Sciences, Federal University of Uberlândia, Uberlândia 38400-732, MG, Brazil; 4 Institute of Biotechnology, Federal University of Uberlândia, Uberlândia 38400-902, MG, Brazil; 5 Chemistry Department, Federal Institute of Triângulo Mineiro, Uberaba 38064-790, MG, Brazil

**Keywords:** antimicrobial activity, biofilm, *Candida* spp., capsaicin, *Capsicum chinense* Jacq., *Toxoplasma gondii*, virulence

## Abstract

Bacterial, fungal, and parasitic infections increase morbimortality rates and hospital costs. This study aimed to assess the antimicrobial and antiparasitic activities of the crude extract from the seeds and peel of the pepper *Capsicum chinense* Jacq. and of the isolated compound capsaicin and to evaluate their ability to inhibit biofilm formation, eradicate biofilm, and reduce hemolysin production by *Candida* species. The crude ethanolic and hexane extracts were obtained by maceration at room temperature, and their chemical compositions were analyzed by liquid chromatography coupled to mass spectrometry (LC–MS). The antimicrobial activity of the samples was evaluated by determining the minimum inhibitory concentration. Inhibition of biofilm formation and biofilm eradication by the samples were evaluated based on biomass and cell viability. Reduction of *Candida* spp. hemolytic activity by the samples was determined on sheep blood agar plates. The antiparasitic action of the samples was evaluated by determining their ability to inhibit *Toxoplasma gondii* intracellular proliferation. LC–MS-ESI analyses helped to identify organic and phenolic acids, flavonoids, capsaicinoids, and fatty acids in the ethanolic extracts, as well as capsaicinoids and fatty acids in the hexane extracts. Antifungal action was more evident against *C. glabrata* and *C. tropicalis*. The samples inhibited biofilm formation and eradicated the biofilm formed by *C. tropicalis* more effectively. Sub-inhibitory concentrations of the samples significantly reduced the *C. glabrata* and *C. tropicalis* hemolytic activity. The samples only altered host cell viability when tested at higher concentrations; however, at non-toxic concentrations, they reduced *T. gondii* growth. In association with gold standard drugs used to treat toxoplasmosis, capsaicin improved their antiparasitic activity. These results are unprecedented and encouraging, indicating the *Capsicum chinense* Jacq. peel and seed extracts and capsaicin display antifungal and antiparasitic activities.

## 1. Introduction

Health care-related infections (HAI) caused by multidrug-resistant bacteria are increasingly common in hospitals worldwide, and ESKAPEEc (*Enterococcus faecium*, *Staphylococcus aureus*, *Klebsiella pneumoniae*, *Acinetobacter baumannii*, *Pseudomonas aeruginosa*, *Escherichia coli*, and *Enterobacter* spp.) has been the most isolated species [1]. HAIs caused by fungal species, especially *Candida* spp., have also increased, significantly impacting morbimortality rates and hospital costs [2].

*Candida* species can produce virulence factors (e.g., hydrolytic enzymes and biofilms) that help initiate and maintain the infectious process [3]. Hydrolytic enzymes degrade host tissues (which facilitates the onset of infection) and help the pathogen obtain nutrients to multiply and propagate [4]. Microorganisms can form biofilms, one of their main defense mechanisms—biofilms make microorganism removal from biotic and abiotic surfaces difficult. They prevent contact between the matrix’s antifungal agents and microbial cells [5]. Production of these virulence factors, associated with indiscriminate use of antimicrobials, has raised the number of infections by resistant fungal isolates, thereby limiting therapeutic options and increasing mortality rates [3].

Parasitic infections are a major public health concern because they impact morbidity rates. Among such infections, toxoplasmosis, a foodborne zoonotic infection caused by *Toxoplasma gondii* [6], stands out. It has severe clinical manifestations in immunocompromised patients, fetuses, and newborns [6,7]. Regarding treatment, sulfadiazine combined with pyrimethamine (SDZ + PYR) is the first choice; however, although this option suppresses active infection, it does not cure latent infection [8]. In addition, these classic drugs are associated with serious maternal and infant side effects and cases of treatment failure, suggesting parasite resistance to SDZ + PYR [9]. In the face of these issues, several studies have reported the antimicrobial, antivirulence, and antiparasitic effects of many natural compounds, and promising results have been published recently [5,10].

The genus *Capsicum* has 35 known pepper species; *Capsicum chinense* is one of the most common species in Brazil and Central America [11]. The chemical composition of peppers belonging to this genus includes phytochemical compounds such as flavonoids, carotenoids, vitamins C, D, and E, and capsainoids, whose medicinal activity has been proven [11,12]. Capsainoids underlie the characteristic pungency of the genus. Capsaicin is the most abundant molecule and has relevant anti-inflammatory and antitumor actions, not to mention that it helps to control cholesterol and obesity [13]. Nevertheless, its antimicrobial [14], antiparasitic [15], and antivirulence [16] activities remain little investigated.

In this study, we aim to evaluate the antimicrobial and antiparasitic activities of the crude extracts from the seeds and peel of the pepper *Capsicum chinense* Jacq. and of the isolated substance capsaicin and to investigate their ability to inhibit biofilm formation, eradicate biofilm, and reduce hemolysin production by *Candida* species.

## 2. Results

### 2.1. MIC, MBC, and MFC Determination

The samples PPHE (Pepper Peel Hexane Extract), PPEE (Pepper Peel Ethanolic Extract), PSHE (Pepper Seed Hexane Extract), PSEE (Pepper Seed Ethanolic Extract), and CPS (capsaicin) did not show antimicrobial action against the investigated bacteria within the evaluated concentration range (0.0115 and 400 μg/mL). However, they displayed antifungal activity, especially against *C. glabrata* (ATCC 2001) and *C. tropicalis* (CI). The seed extracts and capsaicin were the most active (Table 1).

### 2.2. Inhibition of Biofilm Formation

**Figure 1:**
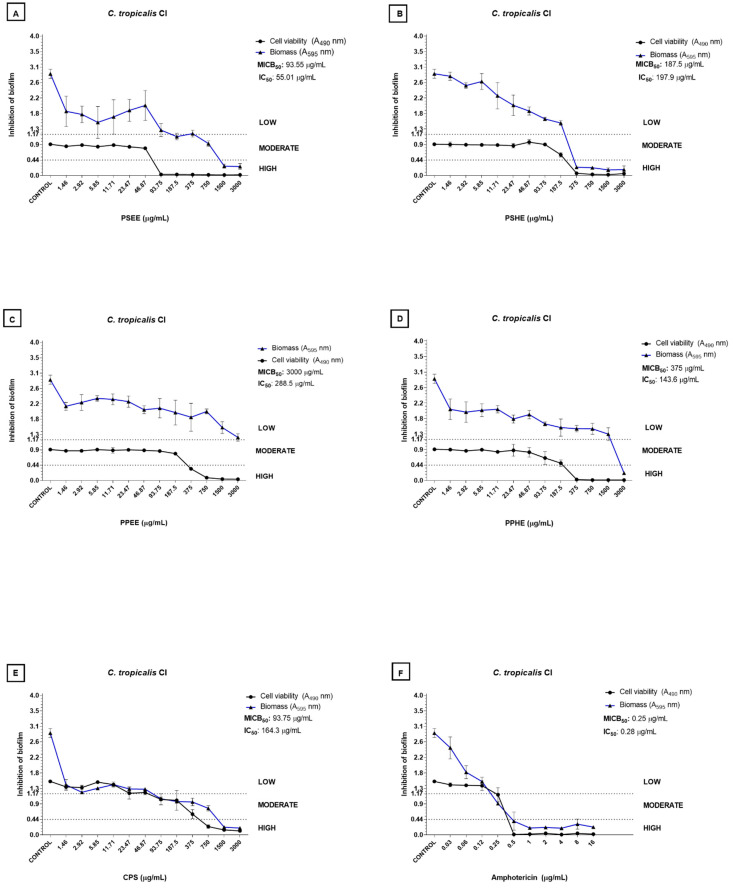
Inhibition of *C. tropicalis* CI biofilm by the hexane and ethanolic extracts from *Capsicum chinense* Jacq. peel and seeds. (**A**–**D**), capsaicin (**E**), and amphotericin B (**F**). The blue line refers to the curve of the OD values of biomass in relation to the concentration of the tested sample. The black line refers to the curve of the OD values of biofilm metabolic activity in relation to the concentration of the tested sample. MICB_50_ values refer to the concentration of the sample that was able to inhibit biofilm formation by at least 50% in relation to the biomass [17]. The IC_50_ values indicate the sample concentration that was able to inhibit biofilm metabolic activity by half compared to the control group [18]. The low, moderate, and high classification refers to biofilm formation inhibition in biomass, where high inhibition corresponds to OD < 0.44, moderate inhibition corresponds to 0.44 < OD < 1.17, and low inhibition corresponds to OD > 1.17. This stratification was based on the classification of *Candida* spp. regarding biofilm formation as proposed by Zambrano et al. [19].

The samples inhibited biofilm formation by *C. tropicalis* (CI) better than the other isolates evaluated. Capsaicin and seed extracts were the most effective (MICB_50_ = 93.75 and 187.5 µg/mL, respectively; Figure 1A,B,E). PSHE at 375 µg/mL inhibited biofilm formation by *C. tropicalis* (CI). As for the reduction in the cell viability of the *C. tropicalis* (CI) biofilm by at least 50%, IC_50_ values varied between 55.01 and 288.5 µg/mL; PSEE provided the lowest value. Finally, 93.75 µg/mL PSEE considerably reduced the number of viable cells in the biofilm (Figure 1).

**Figure 2:**
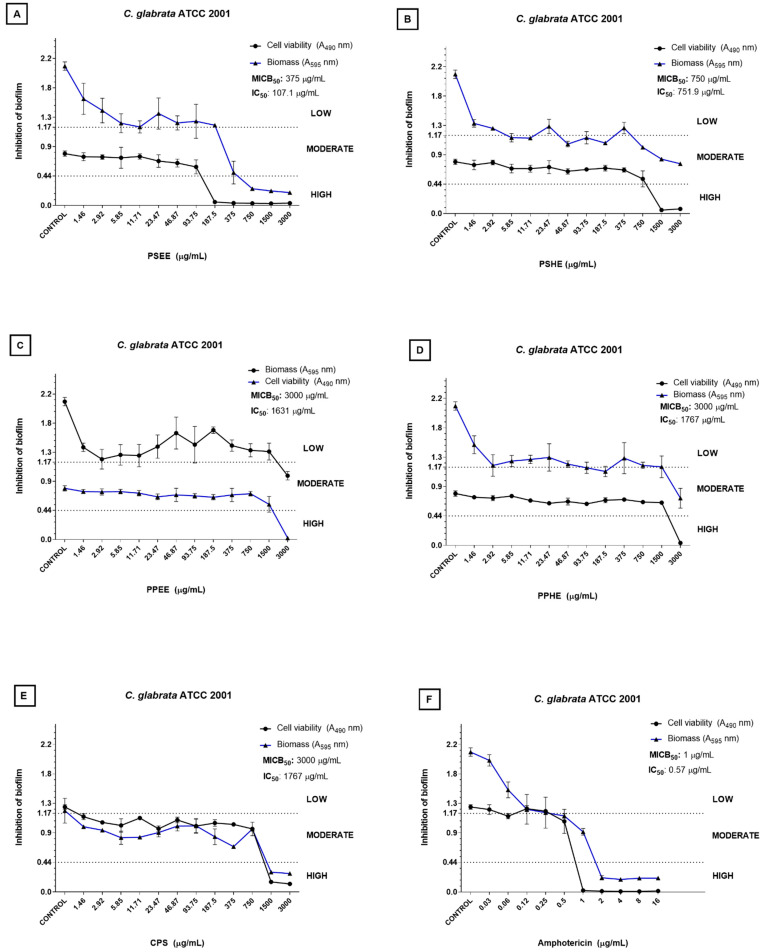
Inhibition of *C. glabrata* ATCC2001 biofilm by the hexane and ethanolic extracts from *Capsicum chinense* Jacq. peel and seeds (**A**–**D**), capsaicin (**E**), and amphotericin B (**F**). The blue line refers to the curve of the OD values of biomass in relation to the concentration of the tested sample. The black line refers to the curve of the OD values of biofilm metabolic activity in relation to the concentration of the tested sample. MICB_50_ values refer to the sample concentration that inhibited biofilm formation by at least 50% in relation to the biomass [17]. The IC_50_ values indicate the concentration of the sample that was able to inhibit biofilm metabolic activity by half compared to the control group [18]. The low, moderate, and high classification refers to biofilm formation inhibition in biomass, where high inhibition corresponds to OD < 0.44, moderate inhibition corresponds to 0.44 < OD < 1.17, and low inhibition corresponds to OD > 1.17. This stratification was based on the classification of *Candida* spp. regarding biofilm formation as proposed by Zambrano et al. [19].

Regarding *C. glabrata* ATCC 2001, PSEE and PSHE gave the best MICB_50_ values: 375 and 750 µg/mL, respectively (Figure 2A,B). Biomass inhibition was high from 750 µg/mL PSEE (Figure 2A). PSEE and PSHE also afforded the best IC_50_ values: 107.1 and 751.9 µg/mL, respectively. Biofilm cell viability inhibition was high from 187.5 µg/mL PSEE (Figure 2A).

### 2.3. Biofilm Eradication

**Figure 3:**
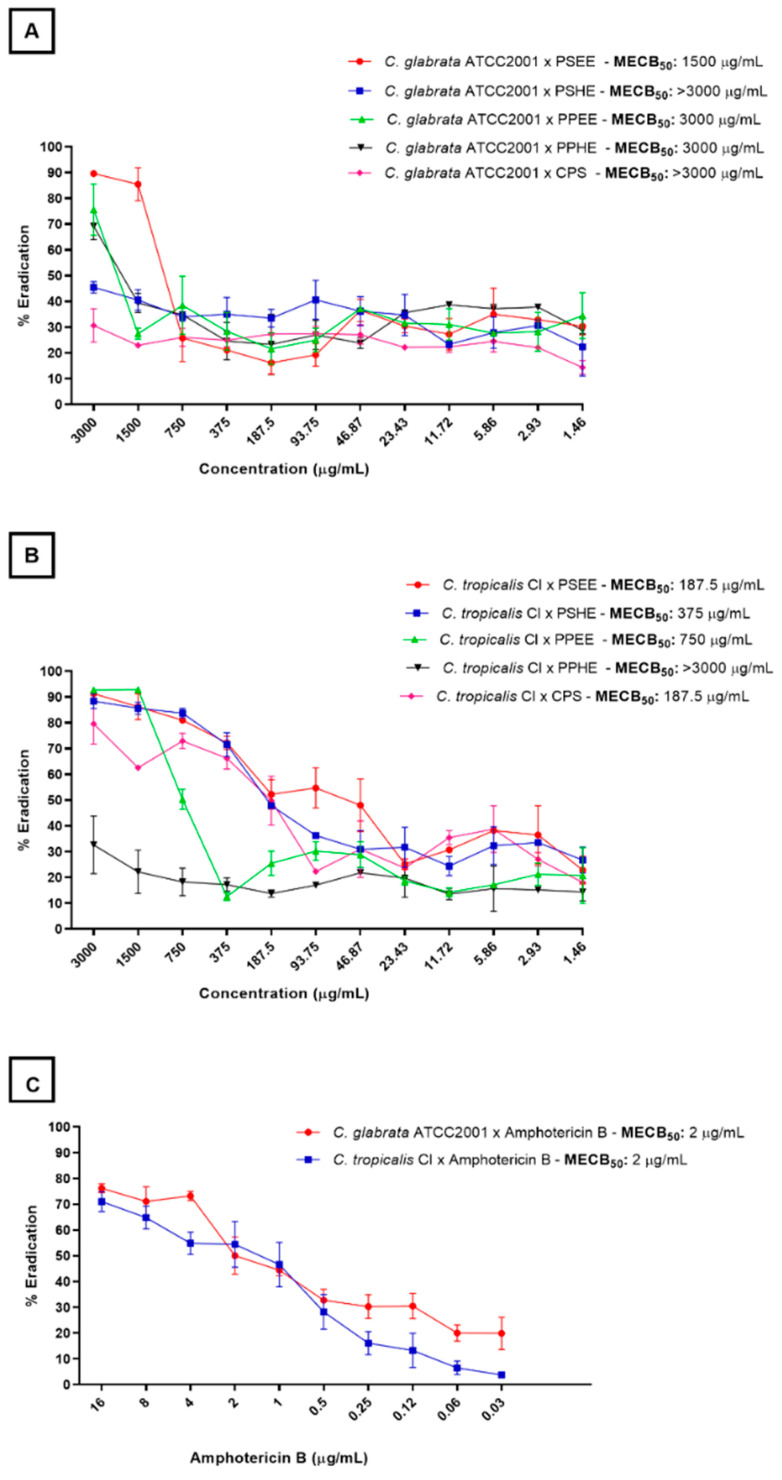
Percentage of viable cell eradication from the preformed *C. glabrata* (ATCC2001) (**A**) and *C. tropicalis* (CI) (**B**) biofilms at different concentrations of the hexane and ethanolic extracts from the *Capsicum chinense* Jacq. peel and seeds, capsaicin, and amphotericin B (**C**). The MBEC_50_ values (Minimum Biofilm Eradication Concentration) refer to the sample concentration that was able to reduce the cell viability of the preformed biofilm by at least 50%.

Figure 3 shows how the ability of the extracts and capsaicin to eradicate the *C. glabrata* (ATCC 2001) and *C. tropicalis* (CI) biofilms varied. The samples effectively reduced the cell viability of the preformed *C. tropicalis* (CI) biofilm. The best samples were CPS and PSEE, which gave MBEC_50_ of 187.5 µg/mL and provided 80% biofilm eradication at the highest concentrations (3000 µg/mL) (Figure 3B). Except for PPHE, all the samples at the highest tested concentrations destroyed over 80% of the preformed *C. tropicalis* (CI) biofilm.

Concerning the preformed *C. glabrata* (ATCC 2001) biofilm, its cell viability decreased significantly when the samples were tested at concentrations above 1500 µg/mL (Figure 3A).

### 2.4. Hemolysin Inhibition

*C. glabrata* (ATCC 2001) exposure to ½ MIC of PSHE, PSEE, and PPHE significantly reduced the hemolytic activity (*p* ≤ 0.05) compared to the control. Treatment with PSEE inhibited this activity by 48.6% (Table 2). As for *C. tropicalis* CI, except for PPEE, all the samples significantly reduced the hemolytic activity. Capsaicin notably resulted in 39.6% inhibition (*p* < 0.0001) (Table 2).

### 2.5. Antiparasitic Action

#### 2.5.1. Cytotoxicity Assay on Host Cells

**Figure 4:**
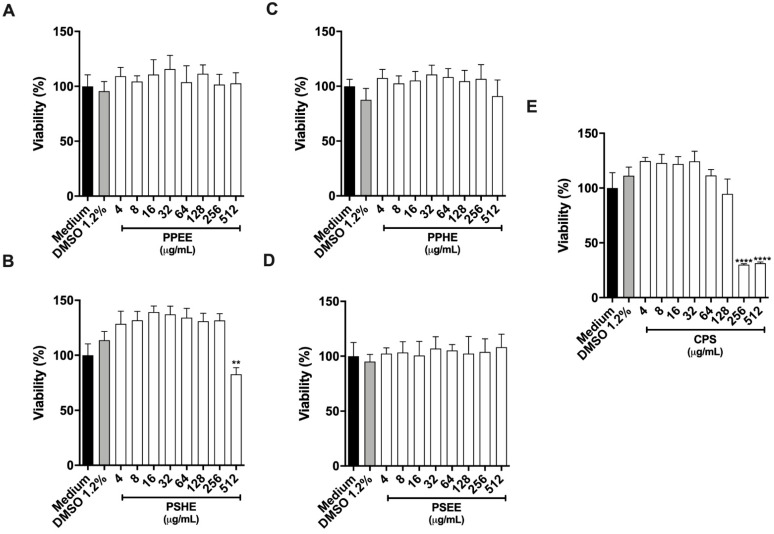
Host cell viability. For 24 h, BeWo cells were treated in twofold serial dilution (ranging from 4 to 512 μg/mL) of pepper peel ethanolic extract (PPEE) (**A**), pepper peel hexane extract (PPHE) (**B**), pepper seed ethanolic extract (PSEE) (**C**), pepper seed hexane extract (PSHE) (**D**), and capsaicin (CPS) (**E**), Cells incubated with culture medium alone (negative control; black column) were considered as 100% viability. Data are expressed as means ± standard deviation. Significant differences detected by the Kruskal–Wallis test and Dunn’s multiple comparison post-test are labeled (statistically significant when *p* < 0.05). ** *p* < 0.01, **** *p* < 0.0001.

BeWo cells lost viability after treatment with high PSHE and CPS doses for 24 h. The minimal dose that elicited the toxic effect was 512 μg/mL for PSHE and 256 μg/mL for CPS as measured by viability loss (Figure 4B,E). PPEE, PPHE, and PSEE did not alter cell viability at any tested concentration (Figure 4A,C,D). BeWo cells treated with 1.2% DMSO did not lose cell viability (Figure 4). The half Cytotoxic Concentration (CC50) against BeWo cells was 144.33 μg/mL for CPS, while for the active extracts, the CC50 was not determined. 

#### 2.5.2. PPEE, PPHE, and PSEE impaired *T. gondii* Intracellular Proliferation in BeWo cells

**Figure 5:**
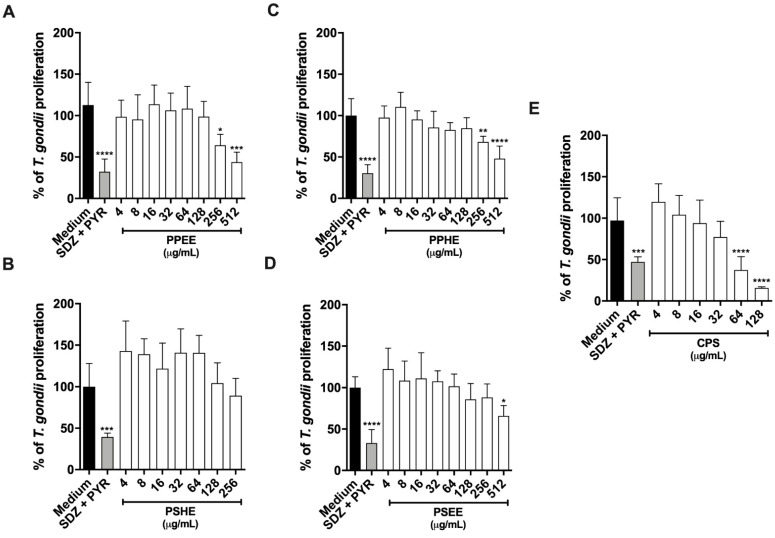
*T. gondii* intracellular proliferation. For 24 h, *T. gondii*-infected BeWo cells were treated with non-toxic concentrations in twofold serial dilutions, as follows: PPEE (**A**), PPHE (**B**), and PSEE (**C**), (all at concentrations ranging from 4 to 512 μg/mL), PSHE (4 to 256 μg/mL) (**D**), and CPS (4 to 128 μg/mL) (**E**), Significant differences detected by the Kruskal–Wallis test and Dunn’s multiple comparison post-test are labeled (statistically significant when *p* < 0.05). * Comparison between infected/untreated cells and infected/treated cells. * *p* < 0.05, ** *p* < 0.01 *** *p* < 0.001, **** *p* < 0.0001.

For 24 h, *T. gondii*-infected BeWo cells were treated with non-toxic concentrations in twofold serial dilutions of PPEE, PPHE, or PSEE (4 to 512 μg/mL), PSHE (4 to 256 μg/mL), or CPS (4 to 128 μg/mL). *T. gondii* intracellular proliferation was quantified by measuring the β-galactosidase activity of viable parasites. All the tested samples inhibited parasite proliferation, as follows: PPEE (256 and 512 μg/mL) (Figure 5A), PPHE (256 and 512 μg/mL) (Figure 5C), PSEE (512 μg/mL), and CPS (64 and 128 μg/mL) (Figure 5E). PSHE was not able to control parasite growth (Figure 5B). The SDZ + PYR treatment reduced parasite proliferation by about 50% compared to the untreated group (Figure 5). The Half Inhibitory Concentration (IC50) against *T. gondii* tachyzoites was 42.12 μg/mL for CPS, with a selective index (SI) of 3.43. The IC50 and SI for the active extracts were not determined. 

#### 2.5.3. CPS Potentiates the action of SDZ + PYR to Control Parasite Growth

**Figure 6:**
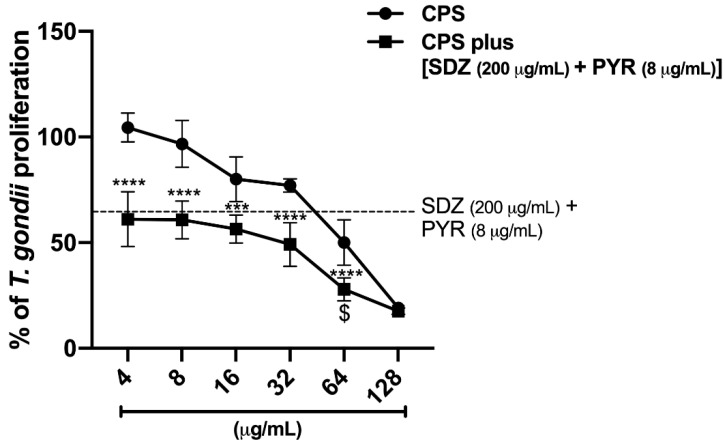
The effects of the association of CPS and SDZ + PYR to control intracellular parasite proliferation. Infected BeWo cells were treated with CPS (4 to 128 μg/mL) alone or in the presence of SDZ + PYR (200 + 8 μg/mL, respectively) for 24 h. Next, *T. gondii* proliferation was quantified by measuring β-galactosidase activity. Significant differences detected by the Kruskal–Wallis test and Dunn’s multiple comparison post-test are labeled (statistically significant when *p* < 0.05). * Comparison between CPS alone with CPS plus (SDZ + PYR). ^$^ Comparison to both CPS alone and SDZ + PYR. ^$^ *p* < 0.05, *** *p* < 0.001, **** *p* < 0.0001.

For 24 h, intracellular *T. gondii* tachyzoites were allowed to grow in BeWo cells in the presence of SDZ + PYR (200 + 8 μg/mL, respectively) alone or in combination with different CPS concentrations (4 to 128 μg/mL). Association of SDZ + PYR with CPS (4 to 128 μg/mL) significantly reduced parasite proliferation compared to CPS alone (Figure 6). In addition, only the combination of SDZ + PYR with 64 μg/mL CPS inhibited *T. gondii* growth significantly more effectively than SDZ + PYR alone and CPS alone (Figure 6).

### 2.6. Analysis of the Chemical Profile of C. chinense Jacq. Extracts by LC-ESI-MS

The samples evaluated in the biological assays (PPHE, PPEE, PSHE, and PSEE) were analyzed by LC-MS-ESI to identify their chemical composition. Table 3 lists the chemical constituents present in the *C. chinense* Jacq. extracts.

Organic and phenolic acids, flavonoids, capsaicinoids, and fatty acids were the main constituents of the ethanolic extracts from peel and seeds. As for the hexane extracts, they contained capsaicinoids and fatty acids as major constituents.

## 3. Discussion

There are no reports on the antimicrobial action of extracts and compounds isolated from *C. chinense* Jacq. However, some studies have already demonstrated the antibacterial potential of molecules isolated from other peppers belonging to the genus *Capsicum* against clinical isolates of *Streptococcus pyogenes* (MIC values between 64 and 128 µg/mL) [16] and *S. aureus* (MIC = 1.2 µg/mL) [14] and standard strains of *Porphyromonas gingivalis* ATCC 33277 (MIC = 16 mg/mL), *Enterococcus faecalis* ATCC 6057 (MIC = 25 µg/mL), *Escherichia coli* ATCC 25922 (MIC = 5 µg/mL) and *Klebsiella pneumoniae* ATCC 29665 (MIC = 0.6 µg/mL) [14]. However, the samples investigated herein do not inhibit bacterial growth in the tested concentration range. The fact that we did not detect antimicrobial action of the extracts and capsaicin against bacterial species, unlike what was reported by other authors, can be explained by the fact that all bacterial isolates evaluated were multiresistant. Perhaps, the way in which the tested compounds prevent bacterial growth is inhibited by some resistance mechanism that the bacteria present.

The antifungal action of extracts and molecules isolated from peppers belonging to the genus *Capsicum*, including capsaicin, has been little reported in the literature. Most studies have been carried out with phytopathogenic fungi, such as *Aspergillus parasiticus* [39] and *Penicillium expansum* [40]. Ozçelik et al. [41] evaluated the antimicrobial action of several *Capsicum* spp. components against standard *C. albicans* ATCC 10231 and *C. parapsilosis* ATCC 22019 strains and found MIC values lower than 16 µg/mL. The discrepancy between these results and the results of the present study might be related to the different types of extracts analyzed in each study and to the origin of the plant material.

However, we have found that PSEE, PSHE, PPHE, and capsaicin present MIC values lower than 200 µg/mL against *C. glabrata* and *C. tropicalis*, which, according to Holetz et al. [42], indicates that these samples have antifungal action. Furthermore, capsaicin exerts a fungicidal action based on the MIC and MFC values. Nevertheless, according to Dorantes et al. [43], the capsaicin mechanism of action remains unknown, but this compound is believed to lyse the cell wall and consequently kill cells.

According to Pappas et al. [2] and Colombo et al. [44], *C. glabrata* and *C. tropicalis* are the most frequent non-albicans species in HAIs in North America, Latin America, and Asia. In addition, *C. glabrata* and *C. tropicalis* isolates present increased fluconazole resistance [45,46], which limits the therapeutic options available for treating invasive candidiasis. Therefore, discovering molecules from natural compounds with promising antifungal action may lead to a therapeutic strategy in the future.

*Candida* spp. produce virulence factors during the infectious process, contributing to worsening patient prognosis [3,5]. In recent years, there has been greater interest in investigating the attributes that contribute to the pathogenicity of this genus and finding ways to inhibit or reduce virulence factor production [5,46,47]. Among virulence factors, biofilm formation plays a crucial role in *Candida* spp. resistance to antifungal agents [5,48]. Thus, developing and using compounds that inhibit biofilm formation should be evaluated as an important therapeutic strategy when treating invasive candidiasis [46].

The results of this study are good, especially concerning the *C. tropicalis* clinical isolate—some of the samples tested here can inhibit biofilm formation by 50% even at sub-inhibitory concentrations. Regarding the preformed biofilm, the concentrations needed for reducing biofilm viability by 50% or more are higher than MICB_50_, which should be expected. As the fungal biofilm matures, its architecture becomes more resistant, making it difficult for compounds with antifungal action to penetrate fungal cells [48]. No literature study has evaluated the ability of extracts from pepper belonging to the genus *Capsicum* and of capsaicin to inhibit biofilm formation or eradicate preformed *Candida* spp. biofilms, so this is the first report in this sense. However, in studies carried out with other extracts and molecules, expressive inhibition of the biofilm formed by *C. tropicalis* clinical isolates has only been possible when concentrations equal to or greater than the MIC were employed [49].

Likewise, at sub-inhibitory concentrations, most samples investigated herein can significantly reduce the *C. glabrata* and *C. tropicalis* hemolytic activity, indicating the antivirulence potential of *C. chinense* Jacq. Nevertheless, no literature study has evaluated the anti-enzymatic action of extracts and molecules from *Capsicum* spp. against *Candida* species, so this is a pioneering study in this sense.

Iron absorption from the lysis of red blood cells may be related to *Candida* spp. resistance to fluconazole [50]. In this context, crude extracts and molecules isolated from natural compounds that have relevant antivirulence action can be evaluated as possible adjuvants in the treatment of invasive infections to reduce the pathogen’s virulence and, consequently, optimize the action of antifungals at lower concentrations.

Despite the relevance of exoenzymes for *Candida* spp. virulence, the action of natural compounds in producing these enzymes remains poorly studied, mainly in relation to hemolysin. Most literature has evaluated compounds’ interference in phospholipase and proteinase production [51].

To assess the anti-*Toxoplasma* activity of the investigated samples, we used human trophoblastic cells (BeWo cells), a well-established in vitro experimental model widely used for studying human congenital toxoplasmosis [10]. PPEE, PPHE, PSEE, and CPS can efficiently control *T. gondii* intracellular proliferation in BeWo cells at concentrations that are not toxic to host cells. This highlights the selective potential of the tested compounds against parasites. Additionally, the CPS antiparasitic activity can be potentialized when it is combined with the classical treatment (SDZ + PYR) against congenital toxoplasmosis.

Piperaceae extracts have distinct pharmacological properties, especially against parasites [52] and tumor cell lines [53]. However, few studies have reported their anti-*T. gondii* activity. Corroborating with our study, Leesombun et al. [54] assessed the effects of ethanolic extracts from Thai piperaceae plants *Piper betle*, *P. nigrum*, and *P. sarmentosum* against infection with *T. gondii* by using in vitro and in vivo models. They demonstrated that *P. betle* is more effective than the other extracts in controlling the parasitic infection in HFF cells and mice [54]. Similarly, the water and ethanol extract from *P. nigrum* and *Capsicum frutescens* can reduce the number of *T. gondii* tachyzoites in the peritoneal fluid of infected mice [55].

Although numerous studies have shown the therapeutic potential of members belonging to the family Piperaceae, most of them have been limited to crude extracts. On the other hand, for the first time, our study has demonstrated the anti-*T. gondii* action of extracts from *C. chinense* Jacq. seeds and peel. We revealed the antiparasitic action of the isolated compound capsaicin using a model of congenital toxoplasmosis. Thus, *C. chinense* Jacq. can be an alternative source of compounds for treating congenital toxoplasmosis, as highlighted using capsaicin, which controlled the *T. gondii* growth rate with low toxicity effects on the host cells.

With respect to the chemical composition of extracts from *C. chinense* Jacq. peel and seeds, LC-ESI-MS analysis, allowed us to identify several classes of metabolites such as organic and phenolic acids, flavonoids, capsaicinoids, and fatty acids. Particularly, organic acids, phenolic acids, and flavonoids occurred only in the ethanolic extracts of *C. chinense* Jacq. peel and seeds. Compounds of these classes of metabolites are known in the *Capsicum* genus, and some of them have already been identified in *C. chinense* fruit [30,56,57].

The organic acids identified in the extracts from *C. chinense* Jacq. peel and seeds include quinic (2), pyroglutamic (3), succinic (4), hydroxyisovaleric (9), hydroxycaproic (13), and 3-phenyllactic (16) acids. Among these acids, quinic (2) and succinic (4) acids have already been determined in the fruit and different parts of the *C. chinense* fruit. We have not found other organic acids such as citric, malic, and fumaric acids reported in *C. chinense* fruits [56,57].

The phenolics protocatechuic (8), *p*-coumaric (17), and ferulic (20) acids identified here have already been reported in the ethanolic extract from *C. chinense* fruits [30]. Genistic, caffeic, and vanillic phenolic acids found in *C. chinense* by Santos et al. [30] were not observed in this study.

Concerning flavonoids, we have identified isoshaftoside (18) in the peel and seed extracts and (iso)orientin (19), vitexin (21), quercetin 3-*O*-rhamnoside (23), and isorhamnetin 3-*O*-rhamnoside (24) in the peel ethanolic extract. Surprisingly, all these compounds are glycoside flavones. Similar results were observed in the study by Materska and Perucka [58] with the *Capsicum annuum* pericarp and fruit, from which several flavonoid glycosides were isolated, including quercetin 3-*O*-rhamnoside (23) identified in *C. chinense* peel.

Some studies with peppers have shown that qualitative and quantitative variations between their constituents may occur [56,57,59]. Some factors such as genetics, cultivar type, maturation stages, irrigation, environmental conditions, soil type, seasonality, extraction processes, and analytical methods can promote these variations [59].

Phenolic acids and flavonoids are related to several biological activities; however, their antifungal actions have been highlighted [60]. Plant extracts rich in phenolic compounds and isolated compounds such as gallic acid (6), protocatechuic acid (8), *p*-coumaric acid (17), ferulic acid (20), and hydroxybenzoic acid (12) exert activity against various *Candida* species [60,61]. In particular, flavonoid aglycones may also contribute to the activity of ethanolic extracts because many of these compounds have antifungal effects alone or in synergistic combination with conventional medicines [62]. Some studies have also shown that flavonoid glycosides alone or in combination are potent anti-*T. gondii* agents [63].

Capsaicinoids are another group of metabolites that can be found in *C. chinense* extracts. Here, we have detected capsaicin (31) and dihydrocapsaicin (34). These two compounds have already been found to be the major capsaicinoids in the fruit of *C. chinense* and other peppers such as *Capsicum annuum* and *Capsicum baccatum* [56].

Capsaicinoids, particularly capsaicin, have been linked to several biological properties, including antibacterial and antifungal effects [13,30,64] and antiparasitic activity [65]. Based on literature data and the results obtained herein for the capsaicin standard, this molecule, together with dihydrocapsaicin (34), may contribute to the activities we have observed for the extracts.

Still concerning the chemical composition of *C. chinense* peel and seeds, they contain several fatty acids that have great antifungal potential, including activity against *Candida* spp. [66,67]. Moreover, the antiparasitic potential of fatty acids has also been evidenced [68]. Therefore, apart from capsaicin, other chemical constituents identified in the extracts may have exerted some effect during the assays.

The chemical composition of *C. chinense* Jacq. peel and seeds agree with the chemical composition of other species belonging to the genus *Capsicum* and other works on *C. chinense*. The classes of metabolites and some identified constituents have already been shown to be active in the biological assays, justifying the promising results found in this study.

## 4. Materials and Methods

### 4.1. Obtaining the Extract and Isolated Substances from the Pepper C. chinense Jacq.

About 2 kg of *C. chinense* Jacq. pepper fruit was collected on 24 February 2020 in the rural area of the municipality of Paranaiguara, Goiás, Brazil. Coordinates: Latitude 18°87′82″ South, Longitude: 50°67′37″ West. Prof. Dr. Jimi Naoki Nakajima kindly identified the material, and a testimonial was deposited at the Uberlandense Herbarium of the Federal University of Uberlândia (UFU) under registration number HUFU 80347. This project was submitted to the National System for the Management of Genetic Heritage and Associated Traditional Knowledge (SISGEN) and is registered under No. A9D4A2D.

After the collection and identification steps, the peel and seeds were separated and placed in a circulating air oven at 35 °C for 7 days for drying. Then, the plant material was ground in a knife mill, and the ethanolic and hexane extracts were obtained by maceration at room temperature, as described by Silva et al. [69]. Briefly, in Erlenmeyer flasks, the material from the peel (0.55 kg) and seeds (0.16 kg) was initially extracted with hexane P.A. (400 mL) at room temperature for 48 h. This procedure was repeated four times. The final volume of hexane that was used in the procedure (1.6 L) was filtered and removed in a rotary evaporator under reduced pressure at 40 °C to give the pepper peel hexane extract (PPHE) and pepper seed hexane extract (PSHE). Subsequently, the remaining plant material was extracted from the peel and seeds with ethanol P.A. (400 mL). The same steps performed for the extraction with hexane were followed, leading to the pepper peel ethanolic extract (PPEE) and pepper seed ethanolic extract (PSEE). The capsaicin used in the study was obtained commercially (Sigma-Aldrich, Darmstadt, Germany) with purity ≥95%.

### 4.2. Analysis by High-Performance Liquid Chromatography Coupled to Mass Spectrometry

The *C. chinense* Jacq. extracts were analyzed by LC-MS on a liquid chromatograph (Agilent, model Infinity 1260) coupled to a high-resolution mass spectrometer QTOF (Quadrupole Time of Flight—Agilent, model 6520 B) with electrospray ionization source (ESI). The chromatographic conditions were Agilent Zorbax C18 column (2.1 mm × 50 mm, 1.8 μm) and ultrapure water with formic acid (0.1% *v*/*v*) (mobile phase A) and methanol (mobile phase B). A volume of 1.0 μL of the sample (2 mg mL^−1^) was injected into the chromatograph. The gradient elution system consisted of 10% B (0 min), 98% B (0–10 min), and 98% B (10–17 min) at a flow rate of 0.6 mL min^−1^. The ionization parameters were nebulizer pressure of 58 psi and drying gas at 8 L min^–1^ at a temperature of 220 °C; energy of 4.5 kV was applied to the capillary. The analysis was performed in the negative mode [M-H]^−^ under high resolution (MS).

The molecular formula was proposed for each compound according to a list sug-gested by the MassHunter Workstation Qualitative Analysis Software Agilent^®^ (Version 10.0) following the smallest difference between the experimental mass and the exact mass, error in ppm, unsaturation equivalence, and nitrogen rule.. The molecular ions’ sequential mass spectrometry (MS^2^) was performed at different collision energies. The chemical composition of the extract was proposed by comparing the obtained mass spectra of the fragments and the mass obtained under high resolution with other works in the literature, Metlin library [20], and PubChem database [24,26,27,28,35,36,37]. The standard capsaicin (Sigma-Aldrich, Darmstadt, Germany) with purity ≥95% in methanol (300 µg mL^−1^) was also analyzed under the same conditions to confirm its presence in the extracts.

### 4.3. Microorganisms

Microbiological assays were performed with standard strains from the American Type Culture Collection (ATCC) and clinical isolates (CI) of multidrug-resistant bacteria and *Candida* species from previous research and maintained at the Antimicrobial Assay Laboratory (LEA-UFU). The assayed microorganisms were *Enterococcus faecalis* (ATCC1299 and CI), *Klebsiella pneumoniae* (ATCC13883 and CI), *Pseudomonas aeruginosa* (48 1997^a^ EPM and CI), *Staphylococcus aureus* (ATCC BAA 44 and CI), *Staphylococcus epidermidis* (ATCC 14990 and CI), *Candida albicans* (ATCC 90028 and CI), *Candida glabrata* (ATCC 2001 and CI), *Candida krusei* (ATCC 6258 and CI), *Candida parapsilosis* (ATCC 22019 and CI), and *Candida tropicalis* (ATCC 13803 and CI). All the isolates belong to the LEA-UFU culture collection and are kept under cryopreservation at −20 °C.

### 4.4. Determination of Minimum Inhibitory Concentration (MIC) and Minimum Bactericidal/Fungicide Concentration (MBC/MFC)

The broth microdilution technique determined the antimicrobial activity of the extracts PPEE, PPHE, PSEE, and PSHE and capsaicin (CPS). This was determined by the broth microdilution technique, as proposed by the Clinical and Laboratory Standards Institute in documents M07-A9 and M27 for assays with bacterial and fungal isolates, respectively [70,71]. MIC, defined as the lowest concentration of the compound capable of inhibiting microorganism growth, was determined.

The final concentrations of the tested samples varied between 0.0115 and 400 μg/mL and 0.98 and 3000 μg/mL in the bacterial and fungal assays, respectively. The antimicrobials amphotericin B (0.031 to 16 µg/mL) and tetracycline (0.0115 to 5.9 µg/mL) and the isolates *C. krusei* ATCC 6258, *C. parapsilosis* ATCC 22019, *E. coli* ATCC 25922, and *S. aureus* ATCC 25923 were used as test controls. The plates were incubated at 37 °C for 24 h and read after 30 µL of 0.02% aqueous resazurin solution was added to observe microbial growth. The development of a blue and pink color indicated the absence and presence of growth, respectively. MIC was the lowest concentration that maintained the blue color in the supernatant medium [72].

Then, 10 µL of the inoculum was removed from each well before resazurin was added to determine MBC and MFC, defined as the lowest concentration of the test sample without any microbial growth. The removed sample was plated with Muller–Hinton agar (bacteria) and Sabouraud Dextrose agar-ASD (yeasts). The presence or absence of growth was observed after incubation at 37 °C for 24 h. The tests were performed in triplicate in independent experiments.

### 4.5. Antivirulence Action Evaluation

Only the isolates that showed promising MIC results according to the classification proposed by Holetz et al. [42] were selected. For plant extracts, MIC values lower than 100 μg/mL, between 100 and 500 μg/mL, from 500 to 1000 μg/mL, and higher than 1000 μg/mL correspond to good antimicrobial activity, moderate antimicrobial activity, weak antimicrobial activity, and inactivity, respectively. Thus, only the samples and isolates for which MIC was lower than 500 μg/mL were selected.

#### 4.5.1. *Candida* spp. Antibiofilm Assay

The ability of PPEE, PPHE, PSEE, PSHE, and CPS to inhibit biofilm formation and eradicate preformed biofilm was evaluated against *C. glabrata* (ATCC2001) and *C. tropicalis* (CI) based on biomass and cell viability.

The tests were performed in 96-well plates, and sample preparation and assay were performed according to the broth microdilution methodology proposed by CLSI [71].

The fungal inoculum was prepared according to Pierce et al. [73]. The final concentration was adjusted to 1 × 10^6^ cel/mL. Then, aliquots of this suspension were added to 96-well plates containing the samples diluted in RPMI-1640 with 2% glucose and buffered with MOPS ([N-morpholino] propane sulfonic acid) to obtain a final concentration ranging from 0.98 to 3000 μg/mL. The plates were incubated at 37 °C for 24 h for biofilm formation and adhesion.

Plates intended for biomass evaluation were processed according to Marcos-Zambrano et al. [19] with modifications. Briefly, after incubation, well contents were gently aspirated, and the plates were washed three times with phosphate-buffered saline (PBS, pH: 7.2) to remove non-adhered cells. Then, the plates were fixed with methanol for 15 min and stained with 0.1% crystal violet solution for 20 min, and the solution was removed after submerging the plates in a container with distilled water. Finally, the adhered crystal was solubilized by adding 33% acetic acid for 30 min, and the absorbance of each well was determined after the plates were read in a spectrophotometer at a wavelength of 595 nm. Thus, it was possible to determine the minimum inhibitory concentration of the biofilm (MICB_50_), defined as the lowest concentration of the sample capable of inhibiting biofilm formation by at least 50% [17].

Plates reserved for evaluating biofilm cell viability were processed according to Pierce et al. [73] and Oliveira et al. [46] with modifications. Then, after incubation, well contents were gently aspirated, and the wells were washed with PBS three times (to remove non-adhered cells). Next, 50 μL of menadione and 2,3-bis (2-methoxy- 4-nitro-5-sulfophenyl)-2H-tetrazolium-5-carboxanilide (MTT) with a final concentration of 0.5 mg/mL were added, and the plates were incubated at 37 °C for 6 h. After that, the formazan product was solubilized by adding 100 μL of dimethylsulfoxide (DMSO) for 10 min, and 80 μL from each well was transferred to another plate and read in a spectrophotometer at a wavelength of 490 nm. Thus, it was possible to calculate the concentration capable of inhibiting the cell viability of the biofilm by 50% (IC_50_) [18].

Then, 100 μL of the isolate suspension with a concentration of 1 × 10^6^ CFU/mL was added to the wells of the plates and incubated at 37 °C for 24 h to assess the ability of the samples to eradicate the preformed biofilm. Then, the non-adhered cells were removed by washing the wells with PBS three times, and aliquots of the samples diluted in RPMI 1640-MOPS were added to obtain a final concentration between 0.98 and 3000 μg/mL. The plates were incubated again at 37 °C for 24 h, and cell biomass and viability were determined as described above. Thus, it was possible to determine the concentration capable of eradicating at least 50% of viable cells from the preformed biofilm (MBEC_50_) [74].

Amphotericin B (final concentration between 0.031 and 16 μg/mL) was used as a test control. Tests were performed in triplicate in independent experiments.

#### 4.5.2. Hemolysin Production Inhibition

The ability of the samples to inhibit or reduce *Candida* spp. hemolytic activity at a sub-inhibitory concentration (½ MIC) was determined as proposed by El-Houssaini et al. [47] and Brondani et al. [75] with modifications. Briefly, from a 24-h culture of the isolate, a suspension was formed in tubes containing PBS with turbidity equivalent to tube 0.5 on the McFarland scale. Then, 500-µL aliquots of this suspension were added to tubes containing 500 µL of RPMI-1640-MOPS plus the test sample so that the final concentrations of the compounds in the tubes were equivalent to the ½ MIC values and that the final amount of fungal cells was 1 × 10^6^ cells/mL in each tube. The material was incubated at 37 °C for 24 h. After this exposure, the tubes were centrifuged at 3000 rpm for 10 min, the supernatant was discarded, and the pellet was washed with PBS and centrifuged again under the same conditions. The procedure was repeated two more times, and the pellet was resuspended in PBS.

Then, 5 µL of this suspension was deposited in equidistant points of Petri dishes containing ASD plus 7% defibrinated sheep blood and incubated at 37 °C for 48 h. The hemolytic activity was evidenced by the presence of a hemolysis halo around the colony. The hemolytic index (Hi) was determined by the ratio between the colony diameter (dc) and the hemolysis halo plus colony diameter (dcp). The results were classified as negative (Hi = 1), moderate (0.63 < Hi < 1), and severe (Hi ≤ 0.63) [76]. Amphotericin B was used as a test control.

The assays were performed in triplicate at two different times. The suspension containing only the RPMI1640-MOPS isolate and broth was used as a positive control, and amphotericin B was used as a control. The test results were expressed as a percentage of inhibition of hemolytic activity according to El-Houssaini et al. [47]. Data normality was verified using the Shapiro–Wilk test. The significance of hemolysis inhibition was determined through analysis by the ANOVA One Way and Kruskal–Wallis test. GraphPad Prisma software, version 8.2, was used, and *p*-values lower than 0.05 were considered significant.

### 4.6. Antiparasitic Effects

#### 4.6.1. Cell Culture and Parasite Maintenance

Human trophoblast cells (BeWo lineage) were commercially purchased from the American Type Culture Collection (ATCC, Manassas, VA, USA). They were maintained in RPMI 1640 medium supplemented with 100 U/mL penicillin, 100 μg/mL streptomycin, and 10% heat-inactivated fetal bovine serum (FBS) in a humidified incubator at 37 °C and in 5% CO_2_. *T. gondii* tachyzoites (highly virulent RH strain, 2F1 clone) constitutively expressing the β-galactosidase gene were cultured as previously described [77].

#### 4.6.2. Host Cell Viability

PPEE, PPHE, PSEE, PSHE, and CPS were solubilized in DMSO and diluted in supplemented RPMI 1640 medium to form a stock solution of 640 μg/mL as previously published [10]. Briefly, BeWo cells (3 × 10^4^ cells/200μL/well) were seeded in 96-well microplates and treated or not with different concentrations of the tested samples (ranging from 4 to 512 μg/mL; twofold serial dilutions) at 37 °C and in 5% CO_2_ for 24 h. Cells were also incubated with 1.2% DMSO (concentration used in the highest treatment dose: 512 μg/mL). We referred to published data to establish the work concentration of sulfadiazine (SDZ) and pyrimethamine (PYR) (200 + 8 μg/mL). The chosen doses have been shown as non-toxic for BeWo cells [78]. BeWo cells were incubated with 5 mg/mL MTT reagent at 37 °C in 5% CO_2_ for 3 h, which was followed by addition of 10% SDS and 0.01 M HCl (37 °C, 5% CO_2_, 18 h,) [79]. Absorbance (570 nm) was measured with a multi-well scanning spectrophotometer. Cell viability was expressed in percentages, with the absorbance of cells incubated with culture medium only considered as 100% viability (Viability %). Dose-response inhibition curves (log (inhibitor) vs. normalized response—variable slope) were obtained using GraphPad Prism Software version 9.3.0.

#### 4.6.3. *T. gondii* Intracellular Proliferation Assay by β-galactosidase Activity

BeWo cells (3 × 10^4^ cells/200 μL/well) were seeded in 96-well microplates. After adhesion, the cells were infected with a highly virulent *T. gondii* strain (RH strain, 2F1 clone) at a multiplicity of infection (MOI) of 3:1 (ratio of parasites per cell) in RPMI 1640 medium containing 2% FBS at 37 °C in 5% CO_2_. After 3 h of invasion, the medium was discarded, and the cells were rinsed with culture medium and incubated at 37 °C and in 5% CO_2_ for 24 h with non-toxic concentrations in twofold serial dilutions, as follows: PPEE, PPHE, and PSEE (4 to 512 μg/mL), PSHE (4 to 256 μg/mL), and CPS (4 to 128 μg/mL). Based on the literature, the present study used the standard treatment with SDZ + PYR (200 + 8 μg/mL, respectively) for comparison with the treatments [78]. The number of tachyzoites was calculated compared to a standard curve produced with free tachyzoites. *T. gondii-*infected BeWo cells incubated with a culture medium in the absence of any treatment were used as negative treatment control (non-inhibited parasite growth) [10,79]. Dose-response inhibition curves (Log (inhibitor) vs. normalized response—Variable slope) were obtained using GraphPad Prism Software version 9.3.0. In addition, the selectivity indexes (SI) were calculated based on the CC50 BeWo cells/IC50 *T. gondii* ratio.

Finally, we assessed whether the anti-*T. gondii* action of CPS could be potentialized in the presence of SDZ + PYR. Briefly, BeWo cells (3 × 10^4^ cells/200 μL/well) were seeded in 96-well microplates and infected with *T. gondii* tachyzoites (3:1) in RPMI 1640 medium containing 2% CO_2_. After 3 h, non-invaded parasites were removed by washing with a culture medium. The cells were treated with CPS (4 to 128 μg/mL) alone or in the presence of a certain concentration of SDZ + PYR (200 + 8 μg/mL, respectively) at 37 °C and in 5% CO_2_ for 24 h. As controls, the cells were incubated with a culture medium only or with SDZ + PYR alone. Finally, *T. gondii* intracellular proliferation was calculated by the β-galactosidase assay, as mentioned above.

## 5. Conclusions

The results of this study are unprecedented and encouraging. We have shown that the extracts from *C. chinense* Jacq. and capsaicin display antifungal action. In addition, they can significantly inhibit the production of virulence factors (such as biofilm formation and hemolytic activity) that are important for the onset and maintenance of invasive candidiasis by *C. glabrata* and *C. tropicalis* thereby indicating their antivirulence potential. Furthermore, we have demonstrated the antiparasitic potential of capsaicin at concentrations that are not toxic to host cells, which attests to the selectivity of this compound toward *Candida* spp. and *T. gondii*. However, studies involving microscopy and molecular assays are needed to elucidate capsaicin’s antifungal and antivirulence action and understand the pathways through which this molecule exerts this effect. In vivo studies for evaluating the toxicity and behavior of capsaicin in the fight against pathogens in the host should also be carried out to confirm the results presented here so that in the future, this compound can be considered a possibility for treating fungal and parasitic infections.

## Figures and Tables

**Table 1:** Minimum inhibitory concentration and minimum fungicide concentration of the hexane and ethanolic extracts from *Capsicum chinense* Jacq. peel and seeds, capsaicin, and amphotericin B against *Candida* spp.

Isolates	Minimum Inhibitory Concentration/Minimum Fungicide Concentration (μg/mL)											
	CPS ^1^		PPHE ^2^		PPEE ^3^		PSHE ^4^		PSEE ^5^		Amphotericin B	
	MIC ^6^	MFC ^7^	MIC	MFC	MIC	MFC	MIC	MFC	MIC	MFC	MIC	MFC
*C. albicans*—ATCC 90028	1500	1500	1500	1500	3000	3000	3000	3000	1500	1500	0.5	0.5
*C. albicans—*CI ^8^	3000	3000	3000	3000	3000	3000	3000	3000	3000	3000	0.25	0.25
*C. parapsilosis—*ATCC 22019	-	-	3000	-	-	-	-	-	1500	1500	0.5	0.5
*C. parapsilosis*—CI	-	-	3000	-	-	-	-	-	3000	3000	0.125	0.125
*C. tropicalis—*ATCC 13803	-	-	3000	3000	3000	-	3000	-	3000	-	0.5	0.5
*C. tropicalis—*CI	187.5	187.5	187.5	750	750	750	187.5	375	93.75	93.75	0.125	0.125
*C. glabrata—*ATCC2001	187.5	187.5	1500	1500	3000	3000	375	375	187.5	187.5	0.25	0.25
*C. glabrata—*CI	1500	1500	1500	1500	3000	3000	1500	1500	750	1500	0.25	0.25
*C. krusei—*ATCC 6258	1500	1500	1500	750	3000	3000	750	1500	3000	3000	1.0	1.0
*C. krusei—*CI	1500	1500	750	750	750	750	187.5	1500	1500	1500	0.06	0.06

Note: ^1^ Capsaicin; ^2^ Pepper Peel Hexane Extract; ^3^ Pepper Peel Ethanolic Extract; ^4^ Pepper Seed Hexane Extract; ^5^ Pepper Seed Ethanolic Extract; ^6^ Minimum Inhibitory Concentration; ^7^ Minimum Fungicide Concentration; ^8^ Clinical Isolate, -: >3000 μg/mL was considered inactive.

**Table 2:** Hemolytic index and percentage of hemolytic activity inhibition for *C. glabrata* (ATCC2001) and *C. tropicalis* (CI) after exposure to ½ MIC of capsaicin, hexane and ethanolic extracts from *Capsicum chinense* Jacq. peel and seeds, and amphotericin B.

*C. glabrata* (ATCC 2001)							
	Control	CPS ^1^	PPHE ^2^	PPEE ^3^	PSHE ^4^	PSEE ^5^	Amphotericin B
Average Hi	0.35	0.47	0.5	0.47	0.48	0.52	0.42
% inhibition	-	34.3%	42.8%	34.3%	37.1%	48.6%	20%
*p* valor		0.13	0.002 *	0.71	0.03 *	0.006 *	>0.99
***C. tropicalis* (CI ^6^)**							
	**Control**	**CPS**	**PPHE**	**PPEE**	**PSHE**	**PSEE**	**Amphotericin B**
Average Hi	0.53	0.74	0.70	0.61	0.67	0.69	0.70
% inhibition	-	39.6%	32.1%	15.1%	26.4%	30.2%	32.1%
*p* valor		<0.0001 *	0.0002 *	0.99	0.0067 *	0.0012 *	0.0004 *

Note: ^1^ Capsaicin; ^2^ Pepper Peel Hexane Extract; ^3^ Pepper Peel Ethanolic Extract; ^4^ Pepper Seed Hexane Extract; ^5^ Pepper Seed Ethanolic Extract; ^6^ Clinical Isolate; * *p* value statistically significant value.

**Table 3:** Compounds identified in PPEE, PPHE, PSEE and PSHE by LC-MS in negative mode.

N.	Rt (Min)	[M-H]^–^	Exact Mass	Error (ppm)	Fragmentos MS^2^	Molecular Formula	Tentative Identity	References
1	0.75	181.0740	181.0745	−2.7	10 eV: 181, 163, 119, 101, 89, 71, 59	C_6_H_14_O_6_	Sorbitol ^1^	[20]
2	0.81	191.0563	191.0561	1.04	10 eV: 173, 158, 127, 109, 93, 85	C_7_H_12_O_6_	Quinic acid ^1,2^	[20,21]
3	0.96	128.0354	128.0353	0.78	10 eV: 128, 112, 99, 88	C_5_H_7_NO_3_	Pyroglutamic acid ^1,2^	[20]
4	1.10	117.0194	117.0193	0.85	10 eV: 99, 73	C_4_H_6_O_4_	Succinic acid ^1,2^	[20,21]
5	1.18	292.1429	292.1435	−2.0	10 eV: 202, 130	C_12_H_22_NO_7_	Fructosyl-leucine/isoleucine ^1^	[22]
6	1.37	169.0143	169.0142	0.59	10 eV: 125	C_7_H_6_O_5_	Gallic acid ^1,2^	[20,21]
7	1.59	164.0724	164.0723	0.60	10 eV: 147, 103, 72	C_9_H_11_NO_2_	Phenylalanine ^1,2^	[20]
8	2.27	153.0194	153.0193	0.65	10 eV: 109	C_7_H_6_O_4_	3,4-dihydroxybenzoic acid (Protocatechuic acid) ^1,2^	[20,23]
9	2.52	117.0561	117.0557	3.41	10 eV: 99, 87, 71	C_5_H_10_O_3_	Hydroxyisovaleric acid ^1,2^	[20]
10	2.73	329.0877	329.0878	−0.30	10 eV: 269, 209, 167	C_14_H_18_O_9_	Dihydroxybenzoic acid methyl ether-*O*-hexoside ^1^	[21]
11	2.92	181.0509	181.0506	1.65	10 eV: 163, 135, 119	C_9_H_10_O_4_	Hydroxyphenyllactic acid ^1^	[20,24]
12	3.33	137.0246	137.0244	1.45	10 eV: 123, 93, 65	C_7_H_6_O_3_	Hydroxybenzoic acid ^1,2^	[20,25]
13	3.66	131.0713	131.0714	−0.76	10 eV: 131, 99, 59	C_6_H_12_O_3_	Hydroxycaproic acid I ^1,2^	[24]
14	4.03	210.0774	210.0772	0.95	20 eV: 163, 124, 94	C_10_H_13_NO_4_	Methoxytyrosine ^1^	[26]
15	4.34	131.0713	131.0714	−0.76	10 eV: 131, 99, 85, 69	C_6_H_12_O_3_	Hydroxycaproic acid II ^1,2^	[27]
16	4.80	165.0559	165.0557	1.21	10 eV: 165, 147, 119, 103, 91, 73	C_9_H_10_O_3_	3-phenyllactic acid ^1,2^	[28,29]
17	4.91	163.0402	163.0401	0.61	10 eV: 147, 119, 103	C_9_H_8_O_3_	2-hydroxycinnamic acid (*p*-coumaric acid) ^1^	[23]
18	5.03	563.1409	563.1406	0.53	20 eV: 563, 503, 473, 443, 425, 383, 353	C_26_H_28_O_14_	Apigenin-6-glucoside-8-arabinoside (Isoshaftoside) ^1,2^	[23]
19	5.27	447.0931	447.0933	−0.44	20 eV: 357, 327, 285	C_21_H_20_O_11_	Luteolin-6-*C*-glucoside or Luteolin-8-*C*-glucoside (Iso)orientin ^1^	[23]
20	5.24	193.0505	193.0506	−0.51	10 ev: 178, 149, 134	C_10_H_10_O_4_	Ferulic acid ^1^	[30]
21	5.46	431.0983	431.0984	−0.23	10 eV: 431, 342, 311, 183	C_21_H_20_O_10_	Apigenin-8-*C*-glucoside (Vitexin) ^1^	[23,31]
22	6.32	187.0974	187.0976	−1.06	5 eV: 187, 125, 97	C_9_H_16_O_4_	Azelaic acid ^1,2,3,4^	[32]
23	6.37	447.0933	447.0933	0.0	20 eV: 343, 300, 301, 271, 227, 179, 151, 109	C_21_H_20_O_11_	Quercetin 3-*O*-rhamnoside ^1^	[20,21]
24	7.01	461.1089	461.1089	0.0	20 eV: 357, 315, 314, 299, 295, 271, 199, 151	C_22_H_22_O_11_	Isorhamnetin 3-*O*-rhamnoside ^1^	[33]
25	8.34	327.2179	327.2177	0.61	20 eV: 291, 211, 171, 137, 85	C_18_H_32_O_5_	Trihydroxyoctadecdienoic acid I ^1,2^	[22]
26	8.49	327.2175	327.2177	−0.61	10 eV: 273, 201, 171, 137, 85	C_18_H_32_O_5_	Trihydroxyoctadecdienoic acid II ^1,2^	[22]
27	8.75	329.2333	329.2333	0.0	20 eV: 329, 311, 293, 275, 229, 211 201, 183, 171, 139, 127	C_18_H_34_O_5_	Hydroxyoctadecanedioic acid I or Trihydroxyoctadecenoic acid I ^1,2^	[21,22,23]
28	8.85	329.2332	329.2333	−0.30	20 eV: 329, 311, 293, 275, 229, 211, 201, 183, 171, 139	C_18_H_34_O_5_	Hydroxy-octadecanedioic acid II or Trihydroxy-octadecenoic acid II ^1,2^	[21,22,23]
29	8.97	329.2334	329.2333	0.30	20 eV: 329, 311, 293, 275, 229, 211, 201, 183, 171, 139	C_18_H_34_O_5_	Hydroxyoctadecanedioic acid III or Trihydroxyoctadecenoic acid III ^1,2^	[21,22,23]
30	9.05	287.2231	287.2228	1.00	20 eV: 287, 269, 241, 211	C_16_H_32_O_4_	Dihydroxy-hexadecanoic acid ^1^	[22]
31	9.12	304.1916	304.1918	−0.65	10 eV: 289, 168, 116	C_18_H_27_NO_3_	Capsaicin ^1,2,3,4^	[34]
32	9.19	329.2335	329.2333	0.60	20 eV: 329, 311, 293, 275, 229, 201, 171, 139	C_18_H_34_O_5_	Hydroxyoctadecanedioic acid IV or Trihydroxyoctadecenoic acid IV ^1,2^	[22,23]
33	9.45	309.2065	309.2071	−1.9	10 eV: 309, 291, 265, 209, 185, 171, 149, 113	C_18_H_30_O_4_	Oxoepoxyoctadecenoic acid I ^1,2^	[35]
34	9.65	306.2072	306.2075	−0.97	20 eV: 247, 170	C_18_H_29_NO_3_	Dihydrocapsaicin ^1,2,3,4^	[20,36]
35	9.76	309.2072	309.2071	0.32	20 eV: 309, 291, 273, 249, 201, 185, 171, 155, 137	C_18_H_30_O_4_	Oxoepoxyoctadecenoic acid II ^1,2^	[37]
36	9.91	311.2230	311.2228	0.64	20 eV: 293, 275, 249, 201, 185, 171, 155, 139	C_18_H_32_O_4_	Dihydroxy-octadecadienoic acid or Linoleic acid hydroperoxide ^1,2,3,4^	[38]
37	10.18	311.2229	311.2228	0.32	20 eV: 293, 275, 249, 201, 185, 171, 155, 139	C_18_H_32_O_4_	Dihydroxyoctadecadienoic acid or Linoleic acid hydroperoxide ^1,2,3,4^	[38]
38	10.56	293.2124	293.2122	0.68	20 eV: 275, 235, 171, 121	C_18_H_30_O_3_	Hydroxyoctadecatrienoic acid I or Oxooctadeca-dienoic acid I ^1,2^	[22,38]
39		291.1968	291.1966	0.68	20 eV: 291, 273, 185, 121	C_18_H_28_O_3_	Oxooctadecatrienoic acid ^3^	[20]
40	10.85	293.2119	293.2122	−1.0	20 eV: 275, 235, 171, 121	C_18_H_30_O_3_	Hydroxyoctadecatrienoic acid II or Oxooctadeca-dienoic acid II ^1,2,3,4^	[22,38]
41		295.2278	295.2279	−0.33	20 eV: 277, 259, 233, 195, 171, 151, 123	C_18_H_32_O_3_	Hydroxyoctadecadienoic acid I ^1,2^	[22]
42	10.90	293.2119	293.2122	−1.0	20 eV: 293, 275, 249, 221, 197, 185, 149, 125	C_18_H_30_O_3_	Oxooctadecadienoic acid III ^1,2,3,4^	[22,38]
43		295.2277	295.2279	−0.67	20 eV: 277, 235, 183, 171	C_18_H_32_O_3_	Hydroxyoctadecadienoic acid II ^1,2,4^	[22]
44	11.86	277.2170	277.2173	−1.0	20 eV: 277, 233	C_18_H_30_O_2_	Octadecatrienoic acid ^3,4^	[21]
45	12.23	279.2331	279.2330	0.35	20 eV: 279, 254, 218, 185, 151, 171, 211	C_18_H_32_O_2_	Octadecadienoic acid ^2,3,4^ (Linoleic acid)	[21]
46	12.58	255.2334	255.2330	−1.56	25 eV: 255, 237, 201	C_16_H_32_O_2_	Hexadecanoic acid ^3,4^ (Palmitic acid)	[21]

Note: Rt = Retention time. ^1^ Pepper Peel Ethanolic Extract; ^2^ Pepper Seed Ethanolic Extract; ^3^ Pepper Peel Hexane Extract; ^4^ Pepper seed hexane extract.

## Data Availability

Not applicable.
